# Canonical and non-canonical functions of the non-coding RNA component (TERC) of telomerase complex

**DOI:** 10.1186/s13578-025-01367-0

**Published:** 2025-03-01

**Authors:** Chongwen Cao, Weiyi Gong, Yuanlong Shuai, Sara Rasouli, Qianyun Ge, Anam Khan, Aleksandra Dakic, Nagireddy Putluri, Gennady Shvets, Yun-Ling Zheng, Danyal Daneshdoust, Rani Mahyoob, Jenny Li, Xuefeng Liu

**Affiliations:** 1 Comprehensive Cancer Center, The Ohio State University, Columbus, OH USA; 2 Biomedical Science Graduate Program, The Ohio State University, Columbus, OH USA; 3 Division of Neuroscience, National Institute of Aging, Bethesda, MD USA; 4 Department of Molecular and Cellular Biology, Baylor College of Medicine, Houston, TX USA; 5 School of Applied and Engineering Physics, Cornell University, Ithaca, NY USA; 6 Department of Oncology, Lombardi Comprehensive Cancer Center, Georgetown University Medical Center, Washington, DC USA; 7 Department of Pathology, Wexner Medical Center, The Ohio State University, Columbus, OH USA; 8 Departments of Pathology, Urology and Radiation Oncology, Wexner Medical Center, The Ohio State University, Columbus, OH USA

**Keywords:** Telomerase, TERT, TERC, Non-coding RNAs, Telomeres, Non-canonical functions

## Abstract

The telomerase complex consists of a protein component (TERT), which has reverse transcriptase activity, and an RNA component (TERC), which serves as a template for telomere synthesis. Evidence is rapidly accumulating regarding the non-canonical functions of these components in both normal or diseased cells. An oligonucleotide-based drug, the first telomerase inhibitor, secured FDA approval in June 2024. We recently summarized the non-canonical functions of TERT in viral infections and cancer. In this review, we expand on these non-canonical functions of TERC beyond telomere maintenance. Specifically, we explore TERC’s roles in cellular aging and senescence, immune regulation, genetic diseases, human cancer, as well as involvement in viral infections and host interactions. Finally, we discuss a transcription product of telomere repeats, TERRA, and explore strategies for targeting TERC as a therapeutic approach.

## Introduction

**Fig. 1:**
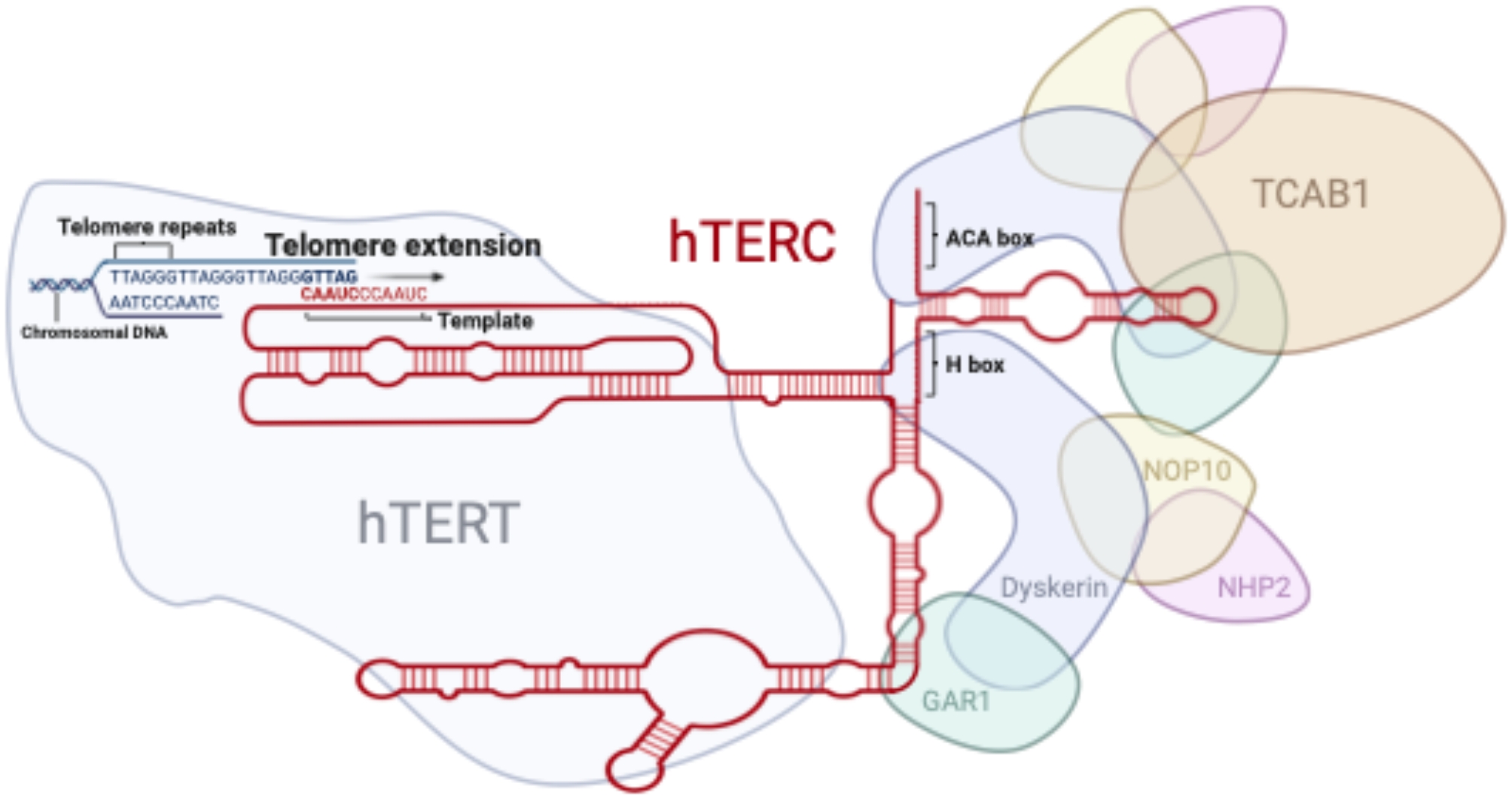
Canonical functions of TERT and TERC. Schematic illustration of the TERC structure and its role in the human telomerase ribonucleoprotein complex, which includes TERT, NOP10, dyskerin, GAR1, NHP2, and TCAB1. TERC provides the template (3’-CAAUCCCAAUC-5’) for hTERT to extend the telomere by adding TTAGGG DNA repeats. The H/ACA box recruits two sets of H/ACA-box-binding proteins, including NOP10, dyskerin, GAR1, and NHP2. TCAB1 is recruited through its binding with TERC and the H/ACA-box-binding proteins

Telomerase is a ribonucleoprotein polymerase (RNP) consisting of a protein component, TERT (1,132 amino acids, 127 kDa), which has reverse transcriptase activity, and an RNA component, TERC (also known as Telomerase RNA Component or TR), which serves as a template for telomere repeats (Fig. 1) [1, 2]. Initially discovered in Tetrahymena extracts, telomerase was described as a specific telomere terminal transferase involved in the de novo elongation of telomeric repeats. The human telomerase was first identified in 1989, and its function is to add the TTAGGG sequence to the ends of telomeres [3, 4]. In addition to the highly conserved TERT and TERC components, several telomerase-associated proteins are required for the functional telomerase complex. These include Ku, HSP90, telomerase-associated protein 1 (TP1), dyskerin (DKC1), telomerase Cajal body protein 1 (TCAB1), non-histone chromosome protein 2 (NHP2), nucleolar protein 10 (NOP10), and GAR1 RNP (GAR1) [5–7]. During early human development, telomerase is active, but its activity becomes silenced between 12 and 18 weeks of gestation, remaining low in somatic cells thereafter. In contrast, most cancer cells and stem cells exhibit relatively high telomerase activity [8–11]. While the mechanisms behind the activation or silencing of telomerase remain to be fully explored, it is widely accepted that there is a direct correlation between TERT expression and telomerase activity, as the TERC component and telomerase-associated proteins are ubiquitously expressed in most human somatic cells [12, 13]. Over the years, research has focused on TERT promoter mutations, gene copy number changes, epigenetic regulation, and transcriptional and post-transcriptional modifications to elucidate the mechanisms behind the switch in telomerase activity [14–22]. It has also been reported that TERC has effects on normal cell biology and disease progression [3, 23, 24].

Substantial research has focused on the role of telomerase in aging, cancer, and disease. Notably, 85% of cancer cells show telomerase activity, highlighting the therapeutic potential of targeting telomerase [9, 25, 26]. Activation of telomerase may also benefit patients with degenerative diseases [27]. However, beyond its canonical role in telomere maintenance, many non-canonical functions of telomerase have been discovered, including its involvement in the regulation of DNA replication and repair, gene expression, and cell signaling pathways. These non-canonical functions may contribute to cell cycle progression, survival, proliferation, differentiation, apoptosis, metabolism, regeneration, and tumorigenesis [22, 28, 29]. Evidence supporting the independent non-canonical roles of TERT and TERC is rapidly increasing [30–32]. For example, quantitative assays for TERT, TERC, and telomerase complexes in HEK293 and HeLa cells reveal approximately 240 telomerase complexes per cell, a number significantly lower than the copies of TERT and TERC. TERC levels often exceed the number of assembled telomerase RNP complexes in cancer cells (approximately 1,150 TERC molecules in HeLa cells, compared to only about 500 molecules of TERT), suggesting the existence of unassembled TERC [30]. Furthermore, the identification of 2,198 TERC-binding sites in the genome presents a substantial resource for studying the potential non-canonical functions of TERC [33]. There are reports of TERC’s alternative functions, independent of telomerase activity, in regulating gene expression and signaling pathways, with subsequent effects on cell survival, apoptosis, inflammation, and cancer promotion [31, 32]. The discovery of TERC-53 has also opened new insights into the non-canonical functions of TERC [34, 35]. Together, these findings underscore the need for further research into the non-canonical functions of TERC in human cell proliferation, differentiation, and disease progression, with significant therapeutic potential.

## Canonical functions of TERC, telomerase inhibitors

TERC is a non-coding RNA that serves as a template for telomere replication by telomerase, enabling the addition of TTAGGG repeats to maintain chromosomal stability. Its canonical role is critical for preserving telomere integrity and ensuring genomic stability, particularly in rapidly dividing cells, such as cancer cells (Fig. 1) [36]. The dependency of telomerase on TERC for its function has made it a key target in cancer research. TERC’s role extends beyond elongating telomeres, as it also stabilizes telomerase and facilitates its recruitment to telomeres [37]. Zhao et al. demonstrated that the absence of TERC (Terc -/-) in embryonic stem cells led to progressive telomere shortening and severe genomic instability across generations. Shortened telomeres disrupted heterochromatin, leading to the activation of retrotransposons like LINE1, which promoted mutations and structural variations, thus underscoring the essential function of TERC in maintaining genomic integrity through telomerase activity [38]. Telomerase inhibitors are designed to suppress the activity of telomerase, targeting either TERC, TERT, or other telomerase-associated proteins [39]. A prominent example is Imetelstat (GRN163L), an oligonucleotide that binds to TERC, preventing its interaction with TERT. By disrupting the canonical function of telomerase, Imetelstat leads to telomere shortening and ultimately triggers apoptosis or senescence in telomerase-dependent cancer cells [40, 41]. In June 2024, the US FDA approved Imetelstat (now branded as Rytelo) for adults with low- to intermediate-risk myelodysplastic syndromes (MDS) and transfusion-dependent anemia who do not respond to erythropoiesis-stimulating agents (ESAs). This approval marked the first telomerase inhibitor to secure FDA approval after 20 years and more than 20 clinical trials across various cancers, including breast cancer, lung cancer, brain cancer, and leukemia [42, 43]. Another approach involves small-molecule inhibitors such as BIBR1532, which directly block the catalytic activity of TERT, impairing telomere elongation. Similarly, stabilizing G-quadruplex structures at telomeres hinders telomerase access, indirectly affecting TERC’s template function [44]. However, these inhibitors face challenges, including potential toxicity to normal stem cells—where telomerase activity is essential—and the development of resistance mechanisms, such as activation of alternative lengthening of telomeres (ALT) pathways [39]. While initial clinical trials for solid tumors showed variable outcomes, promising results have been observed in hematological malignancies, such as myelofibrosis, highlighting the therapeutic potential of targeting TERC in specific cancer contexts [45]. In addition to its canonical role in telomere elongation, TERC has been implicated in genome stability mechanisms [46]. Using a comparative genomic approach, researchers demonstrated that TERC, as part of the telomerase complex, participates in a unique DNA double-strand break repair mechanism [46]. They identified TERC-ITSs (Interstitial Telomeric Sequences), which are sequences retrotranscribed from TERC RNA and flanked by telomeric-like repeats, inserted at break sites during vertebrate evolution. This process involves telomerase retrotranscribing its RNA template into DNA and integrating it into chromosomal loci via the non-homologous end-joining pathway. These findings highlight that, beyond elongating telomeres, TERC plays a broader role in maintaining genome integrity [46]. However, telomerase inhibitors targeting TERC could disrupt these repair mechanisms, potentially increasing genomic instability and raising critical considerations for therapeutic applications [47]. TERC has also been studied as a biomarker, with elevated levels associated with specific cancers, including astrocytoma and gastric carcinomas [48]. Gazzaniga and Blackburn further highlighted the dual role of TERC, demonstrating that its non-canonical function can protect immune cells from apoptosis independent of telomerase activity. Their findings suggest that TERC contributes to immune regulation by mitigating apoptosis through the intrinsic apoptotic pathway, separate from its role in telomere maintenance [32]. Recent research by Majumder et al. underscores the importance of TERC regulation in cancer progression. They identified the RNA-binding protein FXR1 as a critical factor stabilizing TERC in head and neck squamous cell carcinoma [45]. FXR1 binds to G-quadruplex RNA structures within TERC, maintaining its stability and promoting telomerase activity to evade senescence. Silencing FXR1 reduced TERC levels, impaired telomerase function, and induced cellular senescence, linking TERC regulation to the evasion of growth arrest in cancer cells​ [45]. Collectively, these findings emphasize the central role of TERC in telomerase activity, genome stability, and gene regulation. Targeting TERC through direct inhibitors or modulation of its regulatory pathways represents a promising avenue for cancer treatment. However, this requires careful consideration of its multifaceted functions.

## Telomeric repeat-containing RNA (TERRA)

TERRA is a class of long non-coding RNAs transcribed by RNA polymerase II from telomeric and subtelomeric regions of chromosomes. Canonically, TERRA plays a critical role in maintaining genome stability and regulating telomere length [49]. The expression of TERRA varies across different cell types and telomere lengths, with its levels being regulated by chromatin state and cellular stress. In the nuclear foci at chromosome ends, TERRA interacts with telomeric chromatin and associated proteins, such as the shelterin complex. TERRA molecules are G-rich RNA, composed of 5’-UUAGGG-3’ repeats, and exhibit considerable size heterogeneity, ranging from 100 base pairs to over 9 kilobases in mammals [49]. At telomeres, TERRA performs several critical functions. It helps maintain the integrity of heterochromatin by interacting with histone-modifying enzymes, preserving the structure of telomeric chromatin. Beyond its canonical roles, TERRA influences the DNA damage response, gene expression, and certain immune regulatory processes. Moreover, TERRA has been implicated in the development of cancer and inflammatory conditions, some of which occur independently of telomerase activity. These multifaceted roles make TERRA a promising therapeutic target for diseases involving telomere dysfunction and beyond [50, 51].

TERRA also mediates the formation of R-loops, unique three-stranded nucleic acid structures that consist of a displaced single-stranded DNA molecule and an RNA-DNA hybrid. These structures naturally form during transcription and play an important role in telomere maintenance. TERRA-induced R-loops regulate telomere function by recruiting chromatin remodelers and DNA repair proteins [52]. However, when R-loops persist or form inappropriately, they can cause significant genomic instability and DNA damage. This is particularly evident in ALT (alternative lengthening of telomeres) positive cancers, where TERRA-driven R-loops accumulate at short telomeres to facilitate homologous recombination and maintain telomere length. The delicate balance between R-loop formation and resolution is vital for telomere stability [53]. Dysregulation of these structures is increasingly associated with aging and cancer, making them a key focus for therapeutic interventions targeting telomere dysfunction. Understanding the molecular players involved in R-loop formation, resolution, and their interaction with TERRA provides deeper insights into their biological significance and pathological consequences [54].

## Non-canonical functions of TERC

### Cell senescence

Cell aging is a process in which cells gradually lose normal physiological functions, including the ability to proliferate and differentiate [55]. Cellular senescence, a state where cells permanently cease dividing but remain viable, is a key consequence of aging [56]. Normal human cells enter senescence after a limited number of divisions, a phenomenon known as replicative senescence or the Hayflick limit [57]. In addition, cellular senescence can be induced by external or internal stressors such as oxidative stress, UV irradiation, hyperoxia, paracrine signaling from other senescent cells, and DNA damage [58–61]. This type of senescence, known as premature senescence, can occur independently of cell replication. Telomere-related proteins play direct or indirect roles in regulating both replicative and premature senescence.

#### Telomere maintenance, TERRA and replicative senescence

Due to the ‘end-replication problem,’ cells lose a small fragment of telomere repeats with each DNA replication, causing progressive telomere shortening. When telomeres shorten to a critical length, shelterin proteins—including TRF1, TRF2, POT1, RAP1, TIN2, and TPP1—can no longer form the intact shelterin complex, and the t-loop structure is disrupted. This exposes the loose chromosome ends with a single-strand G-rich 3’ overhang, triggering the DNA damage response (DDR) [62, 63]. Research suggests that two major DNA damage-sensing molecules, ATM and ATR kinases, are inhibited by TRF2 and TPP1-POT1, respectively [64]. The telomere DDR further recruits DDR factors, such as 53BP1 and γ-H2AX, and activates downstream p53 signaling, promoting senescence [65, 66].

Telomere length is not shortened at a fixed rate during DNA replication but is instead regulated by several telomere-related proteins. The shelterin complex and telomeric RNA (TERRA) contribute to the formation of secondary structures, including t-loops, R-loops, and G-quadruplexes (G4). These structures can impede telomere replication, requiring specific proteins to resolve them, thus influencing the rate of telomere shortening during DNA replication [67, 68]. Shelterin proteins are crucial for telomere replication. For example, TRF1 is essential for preventing telomere replication fork stalling at telomeres, while TRF2 aids by recruiting Apollo to resolve topological stress [69, 70]. Interestingly, overexpression of TRF1 and TRF2 can stall telomere replication [71]. Additionally, TRF1 and TRF2 help replication forks pass through hard-to-replicate sites in multiple ways. Down-regulation of Timeless significantly accelerates telomere shortening [72, 73]. These results suggest a probably context-dependent manner in whichTRF1 and TRF2 influencing telomere replication. Another shelterin protein, POT1, can promote telomere replication by preventing G4 structures formation [74]. The CST complex also plays important roles with shelterin in protecting telomere ends [75]. A CST complex member, Stn1, regulates DNA polymerase α loading to telomeres [76]. Other non-canonical telomere-related proteins, including helicases and single-strand DNA-binding proteins, contribute to telomere replication. For example, WRN, PIF1, and RTEL1 are involved in unwinding G4 structures at telomeres, while RTEL1 also resolves the t-loop, slowing telomere shortening [77, 78]. RNase H and ATRX can dissolve R-loops by degrading or displacing TERRA [79, 80].

In most normal human somatic cells, telomeres are not lengthened due to undetectable telomerase activity. However, the telomerase complex extends telomeres in stem cells and most cancer cells, preventing senescence. Telomere-related proteins regulate telomerase complex function [81, 82]. For instance, TIN2, a shelterin component, recruits telomerase to the telomere and enhances telomere elongation [83]. Telomerase elongation is facilitated by the inhibition of G4 formation by POT1 [84]. TPP1, another shelterin protein, also recruits telomerase to chromosome ends independently of its protective role [85, 86]. The CST complex, in contrast, competes with POT1 and TPP1 and can act as a telomerase inhibitor [87].

Alternative Lengthening of Telomeres (ALT) is another mechanism to extend telomeres [88]. This pathway is dependent on homology-directed repair (HDR) mediated by RAD51 or RAD52 and regulated by several telomere-related proteins in a context-dependent manner [89, 90]. Shelterin proteins TRF2 and RAP1 inhibit ALT by preventing telomere “ultrabright” formations, which allows HDR factors to localize and form D-loops [91, 92]. Paradoxically, TRF2, RAP1, TIN2, and TRF1 can also support ALT by facilitating the formation of ALT-associated promyelocytic leukemia bodies (APBs) [93, 94]. Furthermore, R-loops promote senescence in some cells by hindering telomere replication and triggering HDR in ALT-positive cells by inducing DSBs at telomeres [79, 80]. This recruits R-loop-regulating proteins such as RNase H, ATRX, RAD51AP1, and Npl3, facilitating ALT-dependent telomere lengthening [88, 95–97]. In ALT-negative cells, RAD51-mediated HDR results in uncapped telomere fusion, leading to stable senescence and genomic instability [98].

These mechanisms play crucial roles in telomere length maintenance during cell proliferation and the onset of replicative senescence.

#### Telomere and premature senescence

Premature senescence can arise from persistent DDR induced by stress [99]. Telomeres are particularly vulnerable to DNA damage and may form persistent DNA damage foci unrelated to replication-induced telomere shortening [100, 101]. Telomere-related proteins regulate stress-induced telomere DNA damage (tDD) and DDR.

Telomeric 8-oxo-guanine (8oxoG), a common damage caused by oxidative stress, activates ATM and ATR signaling to trigger senescence. Acute 8oxoG can even cause telomere loss and crisis [102, 103]. This process detaches approximately 50% of TRF1 and TRF2 from telomeres, leaving telomeric DNA unprotected [104]. MTH1 removes telomeric 8oxoG, thereby protecting telomeres and enhancing telomerase activity [105, 106].

Telomere-related proteins and mitochondrial Functions also play a role in regulating cell aging. In skeletal muscle fibers, TRF2 loss leads to postmitotic cell senescence by downregulating mitochondrial SIRT3 expression, causing mitochondrial dysfunction [107]. Interestingly, FOXO3a can replace TRF2 at telomeres, preventing telomere deprotection [108]. In cancer cells, TIN2 localizes in mitochondria and regulates various metabolic pathways [109]. Additionally, under chronic oxidative stress (such as H2O2 exposure, X-ray irradiation, or excitotoxic glutamate exposure), TERT relocates to the cytosol and mitochondria, protecting mitochondrial DNA while downregulating telomerase activity for telomere elongation [110, 111].

Studies show that telomere damage accumulates persistently during cell aging, suggesting that such damage is difficult to repair [101, 112]. This phenomenon is largely regulated by shelterin proteins. For example, TRF2 inhibits the repair of telomeric single-strand breaks (SSBs). In human fibroblasts, accumulation of stress-induced SSBs drives cellular senescence [113, 114]. DNA double-strand breaks (DSBs) are repaired through classical non-homologous end-joining (c-NHEJ), backup non-homologous end-joining (b-NHEJ), or HDR [115]. However, NHEJ of telomeres increases the risk of chromosomal fusion and requires strict regulation. TRF2 binds to a c-NHEJ factor, Ku, to inhibit telomere NHEJ [116]. TRF2 and RAP1 also inhibit another c-NHEJ factor, DNA-dependent protein kinase, while allowing counteraction with b-NHEJ [117]. Activation of protein phosphatase magnesium-dependent 1 delta (PPM1D) phosphorylates TRF2, enhancing its interaction with TIN2, and promotes the binding of NHEJ factor 53BP1 to telomeres [118]. TRF2-dependent R-loop formation with TERRA recruits RAD52 [119], while telomerase complex protein TCAB1 recruits RNF168, BRCA1, and RAD51, promoting HDR [120].

#### Telomere-related proteins and senescence-related pathways

Telomere is linked to cell senescence not only through the DDR but also via interactions with several key senescence- related pathways. Telomere position effects (TPE) link telomere length to gene expression regulation [121]. It has been shown that telomeres can reach chromosome sites 10 Mb away, enriching TRF2 at the promoter regions of genes and regulating gene expression [122]. In CD4^+^ T cells and promyelocytic cancer cells, TPE was found to affect the expression of TERT [123].

Telomerase complex proteins regulate senescence-related pathways. TERT reduces the ROS level by upregulating GSH: GSSG ratio [124], enhance PINK1 mitochondrial localization, and promote mitophagy [125]. Telomerase complex, including hTERT and TERC, can bind to NF-κB and enhance NF-κB-dependent gene expression [126, 127]. Dyskerin 1 participates in telomerase complex by combining to TERC and interacting with Reptin [128]. Dyskerin 1 loss inhibit ribosome production and lead to p53-mediated cell-cycle arrest [129], while reptin bind to p53 and inhibit its expression [130].

### Immune cell regulation

Mutations in TERC and elevated TERC copy numbers are associated with congenital dyskeratosis, aplastic anemia [131, 132], and other genetic diseases [133–135]. Notably, patients with these conditions often exhibit immunological dysfunction [136, 137]. TERC has been shown to function in immune regulation independent of its telomerase activity (Table 1) [32, 138–140].

**Table 1:** Canonical and non-canonical functions of TERC in Immune Cell Regulation

Disease	Mechanism	Function Type	Reference
Rheumatoid Arthritis	Cytokine regulation via NF-κB pathway.	Non-Canonical	[141]
Multiple Sclerosis	Cytokine regulation via NF-κB pathway.	Non-Canonical	[141]
Type II Diabetes	Cytokine regulation via NF-κB pathway.	Non-Canonical	[141]
Dyskeratosis Congenita	Telomere shortening due to TERC mutations.	Canonical	[142]
Aplastic Anemia	Impaired telomere maintenance in hematopoietic stem cells.	Canonical	[143]
Idiopathic Pulmonary Fibrosis	Telomerase dysfunction in alveolar cells.	Canonical	[144]
Cardiovascular Diseases	Telomere shortening in vascular cells (canonical); oxidative stress regulation (non-canonical).	Canonical & Non-Canonical	[145]
Neurodegenerative Diseases	Telomere maintenance in neurons (canonical); mitochondrial and inflammatory regulation (non-canonical).	Canonical & Non-Canonical	[146]

Gazzaniga and Blackburn [32] investigated TERC’s non-canonical role in immune cell survival, revealing mechanisms that do not depend on its enzymatic activity or telomere elongation. They demonstrated that TERC protects CD4 + T cells from apoptosis induced by dexamethasone, implicating TERC in the intrinsic apoptotic pathway. Knockdown of TERC resulted in activation of pro-apoptotic markers such as Bim and increased caspase-9 and caspase-3/7 activity, confirming TERC’s anti-apoptotic role. Importantly, this function was independent of telomere length or DNA damage, suggesting TERC’s involvement in immune cell homeostasis under stress [32].

Liu et al. [141] further explored TERC’s non-canonical immune-regulatory functions, focusing on its ability to modulate inflammatory responses independently of telomerase activity. They found that TERC upregulates inflammatory cytokines such as IL-6, IL-8, and TNF-α by activating the NF-κB signaling pathway. TERC binds to specific gene promoters (e.g., TYROBP, USP16, TPRG1L, and LIN37) through RNA-DNA triplex formation, enhancing the transcription of these inflammation-related genes. Elevated TERC expression was also correlated with chronic inflammatory diseases such as type II diabetes and multiple sclerosis, where both TERC and its target genes are overexpressed. These findings position TERC as a potential regulator of immune responses and a therapeutic target in inflammation-driven conditions [141].

### Germline mutations in TERC: canonical and non-canonical implications

Germline mutations in TERC gene have been implicated in a spectrum of disorders collectively known as telomere biology disorders (TBDs). These mutations primarily affect telomere maintenance, leading to canonical disease manifestations. However, emerging evidence suggests non-canonical roles for TERC, expanding our understanding of its function in human biology and disease.

#### Canonical implications of TERC mutations

The canonical effects of TERC mutations are primarily related to impaired telomere maintenance. One of the most well-characterized TBDs is dyskeratosis congenita (DC), an inherited bone marrow failure syndrome. Vulliamy et al. first identified heterozygous TERC mutations in autosomal dominant DC, demonstrating that TERC haploinsufficiency leads to telomere shortening and disease [142]. More recently, it has been shown that zebrafish TERC and human TERC (hTERC) can serve as transcription factors that recruit RNA polymerase II, thereby regulating the expression of myeloid genes. This suggests that TERC’s non-canonical functions are closely tied to DC [147]. TERC mutations have also been found in patients with isolated aplastic anemia, highlighting the variable expressivity of TBDs [148]. These findings have expanded the phenotypic spectrum of TERC mutations to include idiopathic pulmonary fibrosis (IPF) and liver disease [149, 150].

The canonical effects of TERC mutations occur through reduced telomerase activity and accelerated telomere shortening. This leads to premature senescence in highly proliferative tissues, which explains the classic triad of mucocutaneous features, bone marrow failure, and cancer predisposition seen in DC [151]. Interestingly, the same TERC mutation can lead to different phenotypes within a family, suggesting that genetic and environmental modifiers influence disease expression [152].

#### Non-canonical implications of TERC mutations

Recent studies have uncovered non-canonical implications of TERC mutations. Beyond its role in telomere elongation, TERC has been shown to influence gene expression through interactions with chromatin-modifying complexes. For instance, Chu et al. demonstrated that TERC can bind to chromatin at non-telomeric sites and regulate the transcription of target genes [153]. This suggests that TERC mutations may affect cellular functions independently of telomere length.

Additionally, TERC has been implicated in mitochondrial function. Sahin et al. showed that telomere dysfunction can impair mitochondrial biogenesis and function through p53-mediated repression of PGC-1α and PGC-1β [154]. Although this study focused on TERC knockout mice, it raises the possibility that TERC mutations in humans might similarly affect mitochondrial homeostasis, contributing to the complex phenotypes observed in TBDs.

TERC’s role in cellular senescence extends beyond telomere shortening. Recent work has demonstrated that TERC can regulate cellular senescence through both telomerase-dependent and -independent mechanisms. For example, manipulating TERC levels through overexpression or depletion affects cellular senescence and proliferation even in telomerase-deficient cells (TERC-/-), suggesting non-canonical functions in senescence regulation [35]. These findings suggest that TERC mutations could impact tissue homeostasis through mechanisms beyond simple telomere attrition, potentially contributing to the diverse phenotypes observed in TBDs.

Understanding both the canonical and non-canonical implications of TERC mutations is crucial for improving the diagnosis and treatment of TBDs. Genetic testing for TERC mutations has become an important diagnostic tool for patients with suspected TBDs [155]. Furthermore, emerging therapies targeting telomerase or telomere biology, such as small molecule telomerase activators or gene therapy approaches, hold promise for treating these disorders [156].

### TERT and TERC in cancer

TERC demonstrates non-canonical functions in cancer, promoting tumorigenesis and progression through mechanisms that are unrelated to telomere elongation. These roles are multifaceted and involve the regulation of gene expression, cellular signaling, and interactions within the tumor microenvironment [157–159]. The role of TERC in cancer development has been particularly elucidated in HPV-related cancers. The E6 and E7 oncogenes from high-risk human papillomavirus (HPV) DNA are present in nearly all cervical cancers, and HPV infection is essential for cancer initiation and development [160]. While E6 and E7 proteins are required for host cell immortalization, they are not sufficient for full cell transformation and tumorigenesis. This suggests that other host factors contribute to malignant transformation and progression. In general, no commonly occurring mutations have been identified in cervical cancer initiation or progression [161]. However, telomerase activity increases in cervical dysplasia due to E6-induced TERT transcription and direct interactions with TERT [162–171]. We and others have shown increased telomerase activity in cells immortalized by the high-risk HPV E6 and E7 oncogenes via three different mechanisms: activation of hTERT, stabilization of hTERT mRNA, and direct interaction with hTERT protein [162–171]. TERT is also increasingly expressed in the cascade of cervical dysplasia [172]. Our previous studies revealed a non-canonical function of TERT in HPV-induced immortalization, where TERT regulates cellular gene expression and HPV promoter activity independent of its telomerase activity [22, 162, 173]. Interestingly, TERC, the RNA component of the telomerase complex, is amplified and overexpressed in nearly all human cervical cancers (> 90%) [174, 175]. Studies have shown that TERC expression is elevated in 20–21% of mild dysplasia (CIN I), 50–68% of moderate dysplasia (CIN II), 81–82% of severe dysplasia (CIN III), and 95–100% in invasive cancers [176, 177]. Recently, we demonstrated that high expression of both TERT and TERC led to increased cell growth, anchorage-independent growth in soft agar, and tumor formation in immunodeficient mice [178]. This represents a key progressive step in the conversion of normal to malignant cells. In cells overexpressing both TERT and TERC, the molecular interplay between HPV oncogenes and telomerase is important for tumorigenesis [178]. These data suggest that TERT and TERC play critical roles in the multistep development of human cervical cancer through both telomerase-dependent and -independent pathways.

Sun et al. [157] uncovered a non-canonical role for TERC in the progression of non-small cell lung cancer (NSCLC). Beyond its role in telomere maintenance, TERC facilitates the nuclear localization of TERT. TERC mediates this process by promoting TERT’s interaction with other telomerase subunits, such as SMG6, in the cytoplasm. This process, supported by the nuclear RNA export factor NXF1, is vital for assembling a functional telomerase complex that maintains telomeres and regulates transcription. Elevated TERC levels, driven by promoter hypomethylation, are associated with more advanced cancer stages and poor patient outcomes [157]. Knockdown of TERC disrupts the assembly and nuclear transport of the telomerase complex, resulting in telomere shortening, decreased NSCLC cell proliferation, and reduced expression of oncogenic factors such as c-Myc, Cyclin D1, and VEGF [157]. These findings highlight TERC as a crucial contributor to NSCLC progression and a promising therapeutic target [157]. ​ Wu and associates [158] reported a non-canonical role for TERC in regulating cell division. The study demonstrates that TERC and the PI3K-AKT signaling pathway are involved in a positive feedback loop. TERC enhances AKT phosphorylation and downstream signaling by transcriptionally activating genes such as PDPK1, a crucial regulator of AKT activation. In turn, activated AKT suppresses the transcription factor FOXO1, which negatively regulates TERC expression, thereby maintaining the feedback loop. This mechanism is implicated in activated CD4 + T cells and normal somatic cells, where TERC-induced AKT activation is essential for cell proliferation. Their research also suggests that TERC overexpression may contribute to tumor progression by upregulating the expression of oncogenic targets such as IL4R and EGFR [158]. Cheng and colleagues [159] identified a novel non-canonical role for TERC in the mitochondria, where RNASET2 converts it into TERC-53 and exports it to the cytosol. Cytosolic TERC-53 levels respond to mitochondrial dysfunction, serving as an indicator of mitochondrial states [159]. This highlights a new mitochondrial retrograde signaling mechanism that connects TERC to cellular regulation and potentially to cancer progression. Jin et al. [179] identified a non-canonical role of TERC in suppressing PD-L1 expression independently of telomerase activity. TERC reduces the stability of PD-L1 mRNA by downregulating the RNA-binding protein HuR, which stabilizes PD-L1 transcripts. Elevated TERC levels accelerate PD-L1 mRNA degradation, inhibiting immune escape mechanisms in cancer cells. The study also demonstrated that the FoxO1 inhibitor AS1842856 could upregulate TERC, counteracting chemotherapy-induced PD-L1 expression, offering potential for combination cancer therapy strategies [179]. Kheimar and colleagues [180] showed that TERC overexpression promotes tumor formation in a virus-induced cancer model. Using recombinant Marek’s disease virus, the researchers replaced the viral telomerase RNA (vTR) with cellular TERC and observed that TERC overexpression restored tumor formation and metastasis in the absence of vTR. This effect was independent of viral replication efficiency, suggesting a telomere-independent mechanism. These findings provide direct evidence that cellular TERC contributes to oncogenesis when overexpressed, highlighting its potential role in virus-induced cancer progression [180].

### Viruses and host interactions

Early reports indicating a reduced papilloma frequency in TERC-deficient cells suggested a potential correlation between TERC and viruses [181]. Subsequent discoveries of virus-encoded telomerase RNA have revealed mechanistic crosstalk between viruses and TERC. For instance, Marek’s disease herpesvirus can encode a TERC-like viral telomerase RNA, which has been shown to promote tumorigenesis [182]. Notably, this function persists even when the viral telomerase RNA is replaced by cellular TERC [180], and both human TERC and viral telomerase RNA exhibit anti-apoptotic effects [183], indicating functional similarities between telomeric and viral RNA.

The correlation between TERC gene amplification and viral signals has been studied in HPV-infected cells. Simultaneous FISH detection of TERC gene copies and integrated HPV in uterine cervix lesions revealed that HPV integration is associated with an increase in TERC gene copy number [184, 185]. Later studies that co-detected TERC lncRNA and HPV E6/E7 mRNA supported this correlation [186]. These studies primarily focused on the co-detection of HPV and TERC as biomarkers for cervical cancer, rather than exploring the underlying mechanisms of this correlation. Furthermore, their findings were mostly limited to lesion samples. However, the development of HPV E6/E7 immortalized keratinocytes has enabled more in-depth in vivo studies on the HPV-TERC interaction. These studies indicated that the simultaneous overexpression of human TERT and TERC could induce tumorigenesis in E6/E7 immortalized cells [178].

Recent research on telomere-associated proteins has also indirectly connected TERC to viruses mechanistically. Dyskerin 1 (DKC1), a telomerase component that binds TERC to modulate telomere maintenance [187], has been shown to pseudouridylate EBER2, an Epstein-Barr virus (EBV)-encoded noncoding RNA, which is crucial for efficient viral lytic replication [188]. Another telomerase component, NHP2, which stabilizes telomeres [189], was found to be upregulated in cells overexpressing the hepatitis B virus X protein (HBx). Knocking down NHP2 inhibited the proliferation of HBx over-expressed cells [190]. TCAB1, a protein that binds and recruits TERC to the Cajal body [191], was also upregulated by EBV to promote the DNA damage response [192]. TPP1, a shelterin component that indirectly binds TERC and recruits telomerase to telomeres [193], is degraded by herpes simplex virus 1 (HSV-1)-encoded E3 ubiquitin ligase [194]. NOP2, a TERC-binding protein associated with catalytically active telomerase [195], has shown essential functions in association with two types of viruses. The HIV-1 Tat protein can be replaced by NOP2 at its binding site on HIV-1 TAR RNA, promoting viral latency [196]. Similarly, HBx has been reported to be post-transcriptionally regulated by NOP2, which adds five-methylcytosine to HBV mRNA, essential for HBV mRNA transportation and HBx translation [197].

Over the past few decades, TRF2, a shelterin component that modulates telomeric maintenance along with TERC [198], has emerged as a key target for multiple types of viruses. The EBV life cycle has been shown to be regulated by TRF2 and other telomeric proteins through modulation of replication at EBV OriP [199]. Later studies revealed that TRF2 is displaced by EBV nuclear antigen (EBNA)-1 [200] and downregulated by EBV latent membrane protein 1 (LMP1) [201]. TRF2 is also post-transcriptionally inhibited during hepatitis C virus (HCV) infection through p53-dependent Siah-1a ubiquitination [202]. In cells infected with human herpesviruses 6 A and 6B, TRF2 is recruited to viral replication compartments (VRCs) to facilitate viral integration [203]. More recently, a study on SARS-CoV-2 infection reported its effect on downregulating TRF2 [204]. Collectively, these findings suggest that TERC shares structural similarities with viral RNA, is correlated with viral latent integration, and overlaps in its reliance on telomere-associated proteins that are crucial for viral life cycles.

### TERC as a target for cancer therapy

In normal somatic cells, telomerase activity is minimal or undetectable, whereas telomerase is frequently upregulated in cancer cells [9]. This makes telomerase a promising therapeutic target for selectively eliminating tumor cells while minimizing adverse effects on healthy tissues.

Small molecule inhibitors and vaccines targeting telomerase are the most common drug candidates in development. The connection between cancer progression and the noncanonical functions of telomerase further strengthens the rationale for targeting telomerase as an anticancer strategy. This approach aims to disrupt the feed-forward regulatory mechanisms that sustain chronic inflammation and oncogenic processes, as well as to mitigate the oncogenic signaling pathways upregulated by telomerase.

Several key signaling pathways involved in tumorigenesis have been linked to telomerase activity, including Wnt/β-catenin [205, 206], NF-κB [205, 207], and DNA damage response (DDR) [208]. For example, the irreversible telomerase inhibitor NU-1 has been shown to impair DNA double-strand break repair by downregulating DDR-related gene expression in the colorectal cancer cell line CT26 [208]. Additionally, novel strategies are emerging to inhibit the interactions between TERT and β-catenin [209] or NF-κB [210], which could complement telomerase-targeting therapies. Further, the non-canonical functions of telomerase components like Reptin and Pontin, which interact with key oncogenic factors such as c-Myc and β-catenin [211–213], also present new targets for cancer therapy. Inhibiting these non-canonical functions of TERC could provide an innovative therapeutic approach to suppress tumor growth. Targeting tumor cell survival and proliferation processes that operate independently of telomere elongation could make cancer therapies more effective. Inhibiting telomerase’s catalytic activity alongside its noncanonical functions may induce senescence in proliferating tumor cells by critically shortening telomeres while simultaneously curbing the activation of oncogenic signaling pathways.

These strategies hold promise for inhibiting telomerase-positive tumors with minimal cytotoxic effects on normal tissues, potentially offering an advantage over current anticancer treatment modalities. Further research into the noncanonical roles of telomerase components, their mechanisms, interacting partners, and potential coactivators is essential for the development of effective therapies targeting telomerase-positive cancers.

One promising strategy involves the use of ligands that induce the formation of quadruplex structures in telomeric DNA, such as telomestatin, RHPS4, TMPyP4, and BRACO-19 [214]. In glioblastoma stem-like cells, RHPS4 has been shown to suppress proliferation independently of telomeric dysfunction, suggesting a differential effect in cancer therapy [215]. However, given that G-rich DNA is abundant throughout the genome, particularly in the promoter regions of oncogenes, the use of G4 ligands may pose risks of off-target effects [216, 217]. TMPyP4, for example, inhibits TERT expression and telomerase activity in humans by downregulating TERT transcription [218, 219].

THIO, 6-thio-dG, also named 6-thio-2’-deoxyguanosine is a nucleotide analog that has the potential to induce telomere dysfunction in telomerase-positive cells by being incorporated into telomeric DNA [220]. In non-small cell lung cancer (NSCLC) xenografts, 6-thio-dG has been shown to inhibit tumor growth by promoting telomere damage [221]. This therapeutic effect has been validated in melanoma [221]. This therapeutic effect has been validated in melanoma [222, 223] and Gliomas [224], both in vitro and in vivo.

Additionally, 6-thio-dG has been found to reduce chemotherapy resistance in EGFR inhibitors. Research demonstrated that NSCLC cells resistant to erlotinib, paclitaxel/carboplatin, and gemcitabine/cisplatin became sensitive to 6-thio-dG treatment [25]. More recent studies have shown that resistance to the EGFR mutant inhibitor Osimertinib in NSCLC involves TERT elevation, and combining Osimertinib with 6-thio-dG induces apoptosis in these resistant cells [225].

6-thio-dG has also shown potential in treating therapy-resistant pediatric brain tumors. It effectively inhibits tumor growth in pediatric high-risk group-3 xenografts from neuroblastoma cell lines and orthotopic patient-derived models of diffuse intrinsic pontine glioma [226, 227]. Additionally, combining 6-thio-dG with anti-VEGF and anti-PD-L1 treatments has significantly improved efficacy in hepatocellular carcinoma [228, 229], particularly through the action of CD8 + T cells.

Using compounds that inhibit TERT DNA-binding sounds less efficient than targeting active TERT sites directly [230, 231]. GRN163L (Imetelstat) binds directly to the TERC component in the catalytic site of the telomerase enzyme inhibiting telomerase [232]. It improves the efficacy of chemotherapy and induce cell death in patient-derived xenografts of Acute myeloid leukemia (AML) [233, 234]. Imetelstat showed a reasonable effect in phase II study of lower-risk myelodysplastic patients [235]. In a phase III study, 40% of patients who are refractory to erythropoiesis-stimulating agents or have relapsed achieved transfusion independence at least 8 weeks [43]. BIBR1532 compound binds to the active sites of TERT non-competitively, inhibiting its telomerase activity [236, 237]. A potential anticancer therapeutic strategy was suggested by Amin et al. who found that cellular proliferation in a zebrafish model was short-term inhibited by BIBR1532 [238]. Supporting this finding, a new BIBR1532-based analogue, demonstrated a stronger anticancer activity in the Ehrlich carcinoma model [232]. The findings suggest that telomerase-targeting therapy exhibits significant potential; however, additional research is necessary to mitigate adverse drug reactions.

Lastly, TERT-derived vaccines have emerged as a promising approach to elicit an immune response against various cancers [239]. Several vaccine candidates are currently in clinical trials, either as single agents [240], or in combination with UV1 [241] or HR2822 [242], and in conjunction with checkpoint inhibitors [243–245]. TERT vaccines have demonstrated substantial therapeutic efficacy in several cancer types through the recognition of CD4+/CD8 + T cells. However, further investigation is required to assess their safety profile and broader applicability across different cancer types [246–248].

## Future aspects

**Fig. 2:**
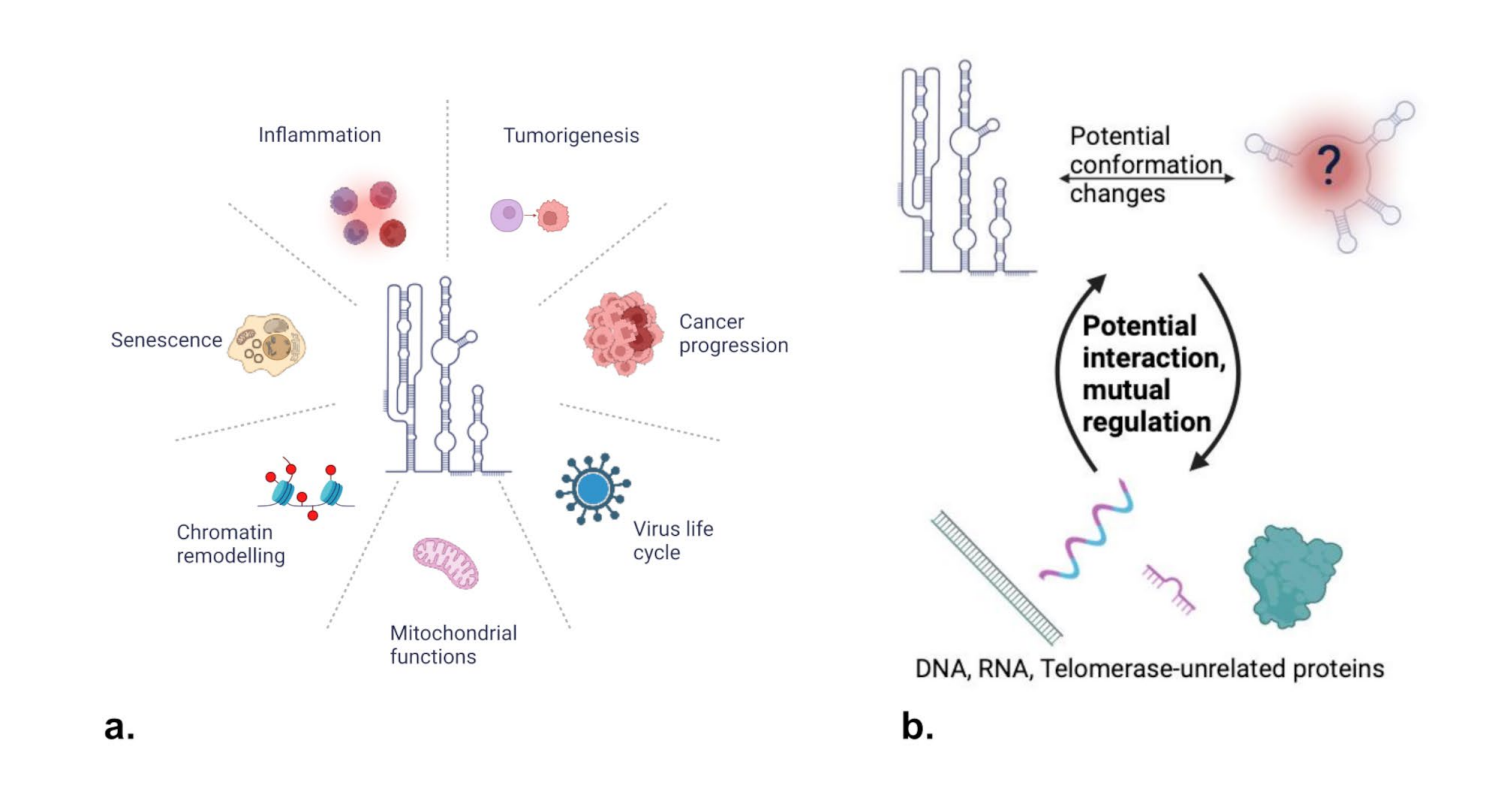
Non-canonical functions of TERC. (**a**) Biological processes that can be regulated by TERC according to current research. (**b**) Schematic illustration of potential mechanisms by which TERC performs known and unknown non-canonical functions

Investigating the non-canonical roles of TERC presents exciting opportunities for advancing our understanding of its functions in cell biology, cancer progression, immune modulation, and therapeutic development. While TERC is widely recognized for its role in telomere maintenance, it is also implicated in various other cellular processes. TERC interacts with numerous molecular pathways, contributing to transcription regulation and chromatin remodeling (Fig. 2) [249]. These interactions are vital for maintaining chromatin stability and ensuring the integrity of the genome. Despite this, the non-telomeric functions of TERC remain relatively underexplored. This gap in knowledge highlights the need for further research to fully map how TERC interacts with proteins and chromatin across different cell types. Recent advances in sequencing techniques are providing high-throughput tools that can significantly enhance our understanding of TERC’s non-canonical roles. For instance, Chromatin Isolation by RNA Purification (ChIRP) has proven effective in enriching RNA-binding proteins and DNA fragments associated with specific long noncoding RNAs, including TERC. Using ChIRP, it was discovered that hTERC binds to several Wnt pathway genes, suggesting a potential regulatory role in cellular signaling [33]. However, additional studies are needed to confirm these findings and elucidate the full scope of TERC’s involvement in these processes. Moreover, a recent study using hTERT ChIP-sequencing has shown that TERT can promote the expression of DNMT3b, a DNA methyltransferase, influencing overall DNA methylation patterns [250]. This finding suggests a possible link between telomerase and epigenetic regulation, but whether these non-canonical functions are dependent on TERC remains an open question.

As research progresses, understanding these alternative functions of TERC could provide valuable insights into its broader role in cellular regulation, cancer progression, and immune response, potentially unveiling new therapeutic strategies.

In cancer, TERC’s role extends well beyond telomere elongation. It may contribute to tumorigenesis through non-canonical pathways, including inflammation, immune system evasion, and genomic instability [251, 252]. These mechanisms, often less explored, could significantly impact tumor progression, especially in cancers that are less reliant on traditional telomerase activity. Understanding these processes could offer a new perspective on TERC’s contribution to cancer biology, potentially reshaping our approach to tumor progression. Beyond its role in cancer development, TERC holds great promise as a diagnostic biomarker. This is particularly true in malignancies where TERC’s non-canonical functions play a more prominent role [253]. Advancements in detection technologies will be pivotal in unlocking TERC’s potential for early cancer diagnosis, providing more accurate and accessible tools for clinicians.

The therapeutic implications of TERC’s non-canonical functions are expansive. In addition to traditional telomerase inhibitors, therapies designed to modulate TERC’s regulatory roles could offer innovative strategies for cancer and other diseases [251]. Potential approaches include small molecules targeting RNA, antisense oligonucleotides, or CRISPR-based tools aimed at modulating TERC expression—either by disrupting or enhancing its function. Furthermore, TERC’s unique role as a regulatory RNA opens the door for engineering TERC for therapeutic purposes. Synthetic versions could be designed to counteract pathological processes or even promote tissue regeneration [254–256]. Integrating TERC-targeted therapies with conventional cancer treatments may also yield synergistic effects, particularly for aggressive or treatment-resistant cancers. This multi-pronged approach could improve treatment outcomes, providing new hope for patients facing challenging diagnoses.

## Data Availability

The authors have nothing to report.
